# Variation in the Early Marine Survival and Behavior of Natural and Hatchery-Reared Hood Canal Steelhead

**DOI:** 10.1371/journal.pone.0049645

**Published:** 2012-11-19

**Authors:** Megan Moore, Barry A. Berejikian, Eugene P. Tezak

**Affiliations:** Manchester Research Laboratory, Northwest Fisheries Science Center, National Oceanic and Atmospheric Administration Fisheries, Manchester, Washington, United States of America; University of Otago, New Zealand

## Abstract

**Background:**

Hatchery-induced selection and direct effects of the culture environment can both cause captively bred fish populations to survive at low rates and behave unnaturally in the wild. New approaches to fish rearing in conservation hatcheries seek to reduce hatchery-induced selection, maintain genetic resources, and improve the survival of released fish.

**Methodology/Principal Findings:**

This study used acoustic telemetry to compare three years of early marine survival estimates for two wild steelhead populations to survival of two populations raised at two different conservation hatcheries located within the Hood Canal watershed. Steelhead smolts from one conservation hatchery survived with probabilities similar to the two wild populations (freshwater: 95.8–96.9%, early marine: 10.0–15.9%), while smolts from the other conservation hatchery exhibited reduced freshwater and early marine survival (freshwater: 50.2–58.7%, early marine: 2.6–5.1%). Freshwater and marine travel rates did not differ significantly between wild and hatchery individuals from the same stock, though hatchery smolts did display reduced migration ranges within Hood Canal. Between-hatchery differences in rearing density and vessel geometry likely affected survival and behavior after release and contributed to greater variation between hatcheries than between wild populations.

**Conclusions/Significance:**

Our results suggest that hatchery-reared smolts can achieve early marine survival rates similar to wild smolt survival rates, and that migration performance of hatchery-reared steelhead can vary substantially depending on the environmental conditions and practices employed during captivity.

## Introduction

Wild and captively-reared salmonids exhibit differences in survival rates [1], [2], behavior [3], [4], morphology [5], [6], and physiology [7], [8]. Some differences, including reduced fitness in at least one hatchery steelhead population [9], reflect effects of domestication selection resulting from adaptation to hatchery environments [10]. Domestication selection in salmon and steelhead hatchery populations may occur during reproduction, early ontogeny, or after release from hatcheries [11]. Recently, some have considered how changes to conventional breeding and rearing practices might reduce the strength of directional selection and minimize deleterious genetic and negative environmental effects of culture [12], [13], but the effectiveness of these types of hatchery reform measures remain largely untested.

Traditional US Pacific Northwest salmon and steelhead hatchery programs for harvest augmentation and mitigation commonly use non-local broodstock and maintain genetically isolated hatchery stocks by intentionally restricting geneflow from wild populations [12]. Hatcheries are increasingly employed to prevent extinction or aid in recovery of depleted salmon populations, including those listed under the U.S. Endangered Species Act. Such programs, referred to as conservation hatcheries, aim to supplement the abundance of naturally spawning fish and conserve genetic resources by quickly amplifying population abundance [14] while attempting to minimize domestication selection or other genetic and ecological risks. Some measures include use of wild (natural-origin), locally-sourced broodstock [15], growth modulation to mimic natural life-history growth patterns [13], [16], avoiding artificial matings and allowing all matings to occur naturally [17], and limiting the duration of the hatchery program to just a few generations [18]. Captively -reared fish are then reintroduced into depleted wild populations at varying life history stages, depending on the program. Much of the research into identifying differences between wild and hatchery salmonids involves fish raised using traditional hatchery methods. Evaluating the performance of fish raised with non-conventional methods is critical to the success and improvement of supplementation and conservation programs [19], [20].

The Hood Canal Steelhead Project (HCSP) is a replicated before-after-control-impact hatchery experiment being conducted in the Hood Canal watershed in Washington State. The HCSP was developed to test the efficacy of using conservation hatcheries to maintain genetic resources and aid in rebuilding of declining wild steelhead populations in Hood Canal. The project began collecting eyed embryos from redds constructed by wild adult steelhead in 2007 and began releasing age-1 hatchery-reared smolts in 2008 and age-2 hatchery-reared smolts in 2009.

The present study used acoustic telemetry technology to assess behavior and estimate survival of hatchery smolts and co-migrating wild smolts as they moved from their natal streams, entered Hood Canal, and migrated to the Pacific Ocean. In one population, the hatchery and wild smolts were offspring of the same breeding populations and therefore allowed us to examine the effects of hatchery and natural rearing environments on migratory behavior and survival without confounding influences caused by genetic effects of past hatchery influences (see [10], [11]). An earlier acoustic telemetry study of steelhead smolt migration [21] estimated freshwater and early marine survival probabilities for one hatchery and four wild Hood Canal populations in 2006 and 2007, tested for factors influencing migration success, and compared travel rates and behavior between populations. This study builds on results from [21] by examining how hatchery fish performance might vary between hatcheries, and comparing survival and behavior of hatchery and wild fish from the same population over three consecutive years.

The early marine phase of the anadromous salmonid life cycle imposes high mortality rates relative to overall marine survival rates [21]–[23], and may be a critical factor limiting the productivity of depleted natural salmon populations. Recent advances in acoustic telemetry technology (e.g., smaller transmitter sizes and better receiver longevity and data capacity) have enabled more detailed, quantitative measurements of juvenile salmonid marine survival and behavior. The present study (a) provides yearly survival estimates for wild smolts from two Hood Canal streams over three consecutive years (2008–2010), (b) tests the null hypothesis that survival rates of wild steelhead smolts do not differ from those of hatchery smolts raised in two different conservation hatcheries, (c) tests the null hypothesis that behavioral traits do not vary between co-habiting wild and hatchery smolts from the same river, and (d) identifies a geographic area within Hood Canal associated with elevated smolt mortality rates for all observed populations.

## Methods

Appropriate scientific collection permits were obtained from the Washington Department of Fish and Wildlife. The study plan was approved by the NOAA Fisheries Northwest Fisheries Science Center. No tagged smolt perished before release as a result of the surgeries performed in this study, and all appeared to be alert, behaving normally, and in good condition upon release.

### Fish Collection and Tagging

**Figure 1:**
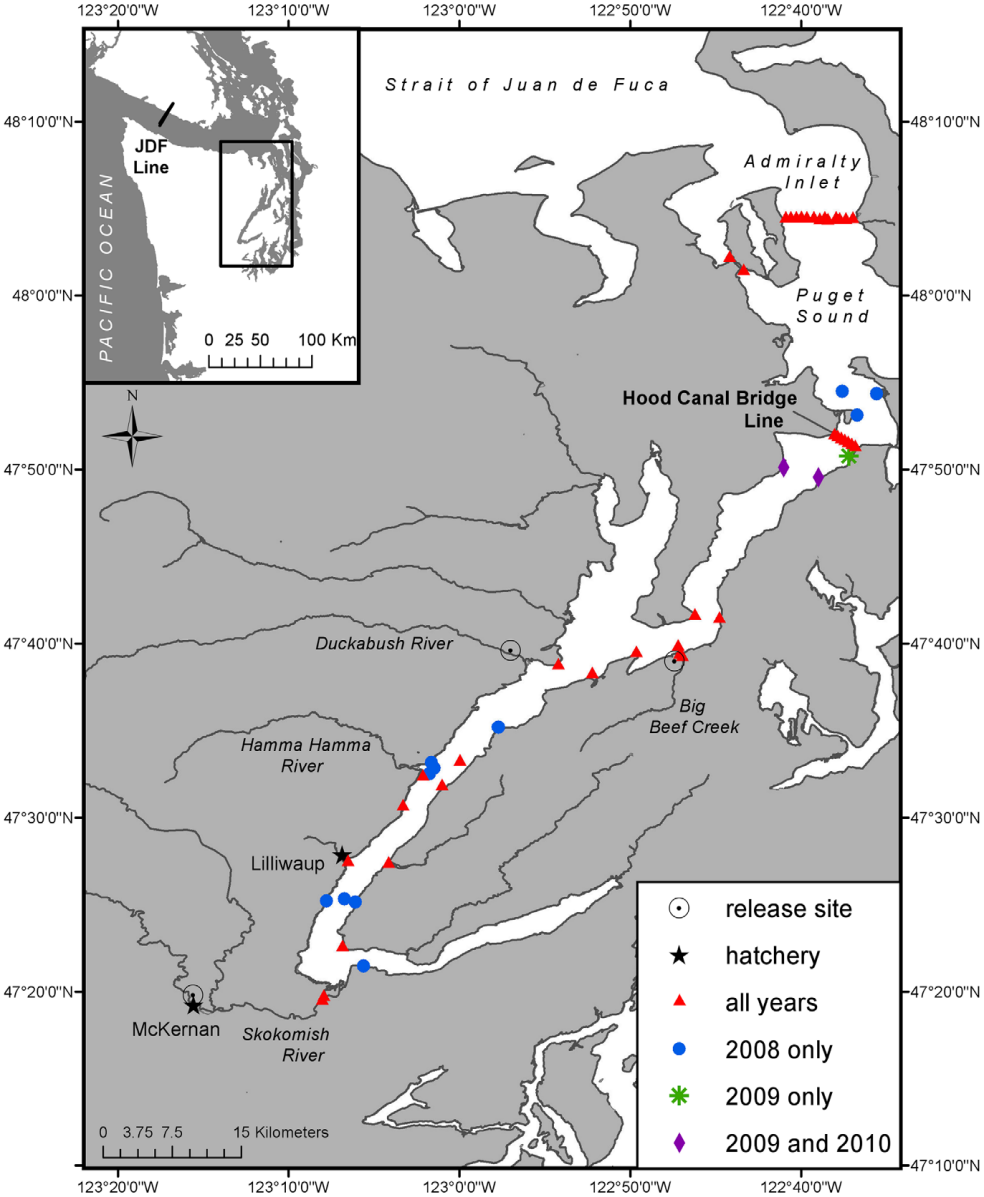
Acoustic receiver locations. In all three study years, one or two receivers were placed at each river mouth to detect outmigrating smolts. The Hood Canal Bridge line was comprised of seven receivers spanning Northern Hood Canal, a line of 33 receivers was deployed across the Strait of Juan de Fuca at Pillar Point, and a line of 13 receivers was deployed in Admiralty Inlet. Additional receivers were placed throughout Hood Canal and Puget Sound to compare migration behavior of wild and hatchery-reared smolts.

Natural-origin steelhead smolts were collected at a weir across Big Beef Creek (n = 95) and a rotary screwtrap in the South Fork Skokomish River (n = 76) during the 2008, 2009, and 2010 outmigration periods (April – June; n = 76; Table 1, Figure 1). Hereafter we refer only to the “Skokomish River” for simplicity, because telemetry receivers were placed in the mouth of the Skokomish River mainstem and the migratory corridor includes both the South Fork and mainstem. To our knowledge, no hatchery steelhead were present in these systems from 2004 through the duration of this study. Hatchery-raised smolts were removed at the eyed egg stage of development from wild steelhead redds in 2007 from the Duckabush River, and in 2007 and 2008 from the Skokomish River, and reared to smolt stage at the Lilliwaup Hatchery (Duckabush River population; n = 30) and the McKernan Hatchery (Skokomish population; n = 101; Table 1, Figure 1), respectively. Fish from both hatcheries were reared in 7.5°–10.5°C fresh water and were hand-fed nearly identical commercially available diets (Table 2). Both hatcheries implemented feeding regimes based on the same temperature-dependent growth model designed to produce growth trajectories similar to natural Puget Sound steelhead (see [13] for rearing details). However, fish reared at McKernan Hatchery (Skokomish population) experienced higher rearing densities than those reared at Lilliwaup Hatchery (Duckabush population). Fish reared at McKernan were size sorted initially after approximately 4 months of rearing, whereas fish reared at Lilliwaup were size sorted a second time after 12 months of rearing (Table 2). Tank geometry also differed between hatcheries, with McKernan fish inhabiting one large raceway during the second year of rearing, while fish at Lilliwaup occupied three circular tanks. Hatchery smolt groups were released back into their river of origin at age-1 (Skokomish: 2008) and at age-2 (Duckabush, 2009; Skokomish: 2009 and 2010). Ideally we would have compared the behavior and survival of wild smolts to hatchery reared smolts of different ages within multiple rivers, replicated over a number of years. However, inefficient screw traps limited our ability to catch a large enough number of wild smolts in the Duckabush River. Therefore, Big Beef Creek wild smolts were used as a surrogate wild population against which a comparison with Duckabush hatchery smolts could be made, because Big Beef Creek enters Hood Canal approximately 1.6 km north of the mouth of the Duckabush River. Therefore, smolts from the two populations experienced very similar marine habitats (Figure 1), though the length of river over which survival was measured did differ (Big Beef Creek freshwater segment = 0.05 km; Duckabush freshwater segment = 1.9 km). The paired evaluations of hatchery and wild smolts in the Skokomish River do provide a direct within-population hatchery-wild comparison of migratory behavior and survival.

**Table 1:** Tagged steelhead smolt physical parameters.

Year	Population	Number Tagged	Mean Fork Length ± SE	Mean Weight± SE	Age at Release	Release Date Range	Mean smolt index	Mean Conditon Factor
2008	Big Beef (W)	27	183±3	57.6±2.6	Mixed	4/16–5/7	2.63	0.93
	Skokomish (W)	41	180±3	53.7±2.5	Mixed	5/2–5/23	2.46	0.97
	Skokomish (H)	42	171±1	49.0±1.1	1	4/29–5/27	2.19	0.90
2009	Big Beef (W)	32	175±2	48.2±2.4	Mixed	4/24–5/10	2.34	0.89
	Skokomish (W)	23	175±4	50.9±3.3	Mixed	4/29/5/27	2.22	0.93
	Skokomish (H)	29	211±3	93.5±3.5	2	4/29–6/6	2.24	0.98
	Duckabush (H)	30	211±2	91.6±3.2	2	5/30–6/6	3.0	0.97
2010	Big Beef (W)	36	178±2	50.2±2.1	Mixed	4/14–5/5	2.80	0.89
	Skokomish (W)	12	167±4	42.6±3.1	Mixed	4/22–5/29	2.45	0.91
	Skokomish (H)	30	201±3	76.3±3.6	2	4/22–4/30	1.77	0.93

**Table 2:** Hatchery conditions and practices.

	Lilliwaup Hatchery(Duckabush)	McKernan Hatchery(Skokomish)
Mean water temperature	8.9°C	8.6°C
Mean density index (ponding to age-1)^a^	0.0066 (6.30 × 10^−5^)	0.290 (0.185)
Mean density index (age-1 to age-2)^a^	0.0065 (9.03 × 10^−6^)	0.056 (0.101)
Vessel configuration (ponding to age-1)	10′ circular	16′ circular
Vessel configuration (age-1 to age-2)	20′ circular	12 × 140′ raceway
Number of size sorts (ponding to age-1)	2	1
Number of size sorts (age-1 to age-2)	0	0
Feeding frequency	3×/d, 4 days/wk	2–3×/d, 7 days/wk
Feed Manufacturer	Bio-Oregon	Bio-Oregon

a Fish densities are expressed in lbs/ft^3^ divided by the average fish length in inches [50] and kg/m^3^/mm in parentheses.

VEMCO V7 transmitters (V7-2L-R64K 7 mm diameter × 17.5 mm length, 1.4 g weight, 69 kHz frequency, 30–90 s ping rate, VEMCO, Ltd., Halifax, Nova Scotia) were surgically implanted in each smolt. For details of the surgical tagging protocol, see [21].Tagging of wild smolts occurred at the smolt collection locations on Big Beef Creek and the Skokomish River. Skokomish hatchery smolts were transported to the smolt trapping location on the Skokomish River, tagged there, held for at least 24 hours, and released. Wild smolts were also collected, held, tagged, and released 24 hours later. Duckabush hatchery smolts were tagged at the Lilliwaup Hatchery, held for 24–96 hours, transported to the Duckabush River, and released.

### Receiver Arrays

Four main acoustic receiver arrays and several individual receivers were deployed to detect tagged smolts at critical points during seaward migration. Two VEMCO VR-2 receivers were deployed at the mouth of the Skokomish River and Big Beef Creek, and one receiver was deployed near the mouth of the Duckabush River to estimate survival from the point of release (PR) to each river mouth (RM). Seven VR-2 receivers were suspended at regular intervals (average of 330 m) across the Hood Canal Bridge (HCB) to detect passage through the northern end of Hood Canal in 2008 and 2010. In 2009, the east half of the Hood Canal Bridge was being replaced, therefore only four receivers were suspended from the west half of the HCB and two receivers were deployed 1 kilometer south of where the bridge is normally anchored (Figure 1). An array of 13 VR-2 receivers was deployed across Admiralty Inlet (ADM) in 2008, 2009, and 2010 to detect smolts passing through northern Puget Sound. A final line of 31 VR-2 receivers spanned the Strait of Juan de Fuca (JDF) at Pillar Point (2008–2010) to detect smolts migrating out to the open ocean (Figure 1). The ADM and JDF lines were deployed and maintained by the Pacific Ocean Shelf Tracking Project (http://www.postcoml.org). Twenty-five (2008), 16 (2009), and 15 (2010) additional VR-2 receivers were deployed throughout the Hood Canal to monitor migration behavior each year (Figure 1).

### Survival Analysis

Cormack-Jolly-Seber (CJS) mark-recapture methodology [24] was used to estimate apparent survival probabilities (φ) from PR-RM (0.05–13.5 km), RM-HCB (24–75 km), HCB-ADM (25 km), and ADM-JDF (110 km), and detection probabilities (*p*) at the four major receiver lines (RM, HCB, ADM, and JDF; Figure 1). The R (R Development Core Team 2007) package RMark [25] was used to construct φ and *p* models for the program MARK [26]. Models incorporated data from all 302 tagged individuals. Goodness-of-fit of the detection data to the CJS model was tested using the program RELEASE (within MARK) and the variance inflation factor was found to be satisfactory (ĉ = 1.302). One important issue with the CJS model is the inability to distinguish between mortality and emigration, so in this study, 1- φ represents both animals that died and those that did not migrate. This issue generally tends to cause underestimation of survival.

Unique combinations of grouping and continuous variables were used to construct a series of models to be tested in RMark. Akaike’s Information Criteria (AIC) were used to identify the set of variables that parsimoniously explained the variation in the survival and detection data [27]. Modeling results were adjusted using the estimated variance inflation factor (ĉ) to compute QAIC_c_ values, which are adjusted AIC values that compensate for extra-binomial variation and small sample sizes. Though testing all combinations of variables produces a large number of models to consider, this method has been deemed optimal [28], and thus was executed to determine the model for both *p* and φ with the lowest QAIC_c_ (Table 3). The detection probability portion of each model was parameterized to represent varying *p* at each river mouth (RM:line) and shared *p* at remaining lines to account for the initially unique migration routes taken by each population. Year (factor) and release date (rd; covariate) were tested as additional sources of variation in detection rate (Table 3). Five factors representing population groupings were compared to the constant and time-dependent survival models; (1) “population” estimated different φ for each population, (2) “rearing type” estimated different φ for hatchery populations and wild populations, (3) “hatchery” estimated different φ for wild populations, the population raised at Mckernan hatchery (Skokomish), and the population raised at Lilliwaup Hatchery (Duckabush), (4) “SkokH” jointly estimated φ for wild and Duckabush hatchery populations, and estimated separate φ for the Skokomish hatchery population, and conversely (5) “DuckH” jointly estimated φ for wild and Skokomish hatchery populations, and estimated separate φ for the Duckabush hatchery population. These factors, designed to test the hypothesis that either or both hatchery populations survived similarly to wild smolts, were individually modeled either linearly or multiplicatively in relation to the segment variable either with or without a “year” factor. Covariates tested for their effect on φ included length (L), condition factor (K; weight/length^3^), and release date (rd) (Table 3).

**Table 3:** Program MARK detection and survival probability modeling results (top 35 models shown).

Model	Number of parameters	QAICc^a^	ΔQAIC_c_	Weight
φ(segment × SkokH + rd), p(∼segment + RM:line)	15	791.771	0	0.160
φ(segment × SkokH + rd), p(segment + RM:line + rd)	15	791.889	0.118	0.150
φ (segment × SkokH + rd + length + k), p(segment + RM:line + rd)	17	794.047	2.277	0.051
φ (segment × SkokH + year + rd), p(segment + RM:line + rd)	17	794.441	2.670	0.042
φ (segment × SkokH + year + rd), p(∼segment + RM:line)	17	794.560	2.780	0.039
φ (segment × SkokH + rd), p(segment + RM:line + year)	17	794.960	3.190	0.032
φ (segment × SkokH + rd + length + k), p(∼segment + RM:line)	17	795.074	3.303	0.031
φ (segment × reartype + rd + length), p(segment + RM:line + rd)	16	795.391	3.620	0.026
φ (segment × SkokH + rd + length), p(∼segment + RM:line)	16	795.687	3.916	0.023
φ (segment × reartype + rd + length), p(∼segment + RM:line)	16	795.728	3.957	0.022
φ (segment × SkokH + rd + length), p(segment + RM:line + rd)	16	795.815	4.044	0.021
φ (segment × reartype + rd), p(∼segment + RM:line)	15	796.062	4.291	0.019
φ (segment × hatchery + rd), p(∼segment + RM:line)	19	796.262	4.492	0.017
φ (segment × SkokH + rd + length), p(segment + RM:line + year)	18	796.288	4.518	0.017
φ (segment × SkokH + year + rd + length), p(segment + RM:line + rd)	18	796.311	4.540	0.017
φ (segment × reartype + rd), p(segment + RM:line + rd)	15	796.352	4.582	0.016
φ (segment × SkokH + rd), p(segment + RM:line + rd + year)	18	796.383	4.613	0.016
φ (segment × hatchery + rd), p(segment + RM:line + rd)	19	796.383	4.613	0.016
φ (segment × SkokH + year + rd), p(segment + RM:line + year)	19	796.453	4.683	0.015
φ (segment × SkokH + year + rd + length), p(∼segment + RM:line)	18	796.466	4.695	0.015
φ (segment × reartype + rd), p(segment + RM:line + year)	17	796.940	5.170	0.012
φ (segment × hatchery + rd + length), p(∼segment + RM:line)	20	797.181	5.411	0.011
φ (segment × hatchery + rd + length), p(segment + RM:line + rd)	20	797.246	5.475	0.010
φ (segment × reartype + rd + length + k), p(∼segment + RM:line)	17	797.399	5.628	0.009
φ (segment × reartype + rd + length + k), p(segment + RM:line + rd)	17	797.759	5.989	0.008
φ (segment × SkokH + year + rd), p(segment + RM:line + rd + year)	20	798.026	6.255	0.007
φ (segment × SkokH + rd + length + k), p(segment + RM:line + year)	19	798.158	6.388	0.007
φ (segment × SkokH + year + rd + length + k), p(segment + RM:line + rd)	19	798.345	6.574	0.006
φ (segment × SkokH + year + rd + length), p(segment + RM:line + year)	20	798.414	6.644	0.006
φ (segment × hatchery + year + rd), p(segment + RM:line + rd)	21	798.460	6.689	0.006
φ (segment × SkokH + year + rd + length + k), p(∼segment + RM:line)	19	798.462	6.691	0.006
φ (segment × hatchery + year + rd), p(∼segment + RM:line)	21	798.555	6.785	0.005
φ (segment × SkokH + year), p(segment + RM:line + rd)	16	798.618	6.848	0.005
φ (segment × SkokH + year), p(∼segment + RM:line)	16	798.643	6.872	0.005

a QAICc = Akaike’s Information Criterion adjusted for extra-binomial variation and small sample sizes.

The CJS model uses detections at subsequent encounter occasions to estimate *p* for each previous occasion; therefore, φ and *p* are confounded for the last receiver line. To circumvent this problem, empirically derived estimates from similarly sited and configured receiver lines were used to fix *p* at the JDF line [29]. Melnychuk [30] calculated mean and 95% confidence limit estimates of *p* for V7 VEMCO tags passing a receiver line spanning the Strait of Georgia in 2004, 2005, 2006, and 2007, so we used an average of the 2005–2007 values (2004 was an anomalous year) for all years to fix the value of *p* for the JDF line in our models (*p_JDF,_*_fixed_ = 0.685). To deal with the uncertainty associated with fixing the detection probability at the JDF line, we also calculated survival estimates with *p* fixed at the above mentioned lower and upper 95% confidence limit values (0.428 and 0.863, respectively) to obtain a range of values for φ. For the remaining parameters, estimates of φ and *p*, and standard errors (based on the model’s variance-covariance matrix) around those estimates were derived from the model with the lowest QAIC_c_ (Table 4). Distance-based mortality rates (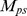) were calculated using the expression:

**Table 4:** Segment-specific per cent survival probabilities ± standard error derived from the model with the lowest QAIC_c_ that included year (φ(segment × SkokH + year + rd), *p*(segment + RM:line + rd)).

Year	Population	PR^a^-RM^b^ (FW)	RM-HCB^c^	HCB-ADM^d^	ADM-JDF^e^	RM-JDF (Marine)	RM-JDF range^f^
2008	Big Beef Wild +Skokomish Wild	96.9±2.2 (37)	89.2±6.3 (52)	38.9±8.0 (15)	45.7±12.3 (6)	15.9±5.2	12.2–25.9
	Skokomish Hatchery	58.7±12.0 (10)	34.1±10.8 (7)	47.0±18.7 (5)	32.0±25.1 (1)	5.1±10.8	3.8–8.9
2009	Big Beef Wild +Skokomish Wild +Duckabush Hatchery	96.1±2.9 (57)	86.4±8.0 (48)	32.9±7.0 (21)	39.3±11.6 (9)	11.2±2.6	8.4–18.7
	Skokomish Hatchery	52.2±12.5 (6)	28.5±10.3 (17)	40.6±18.3 (2)	26.6±22.6 (1)	3.1±5.3	2.3–5.2
2010	Big Beef Wild +Skokomish Wild	95.8±3.0 (38)	85.5±8.3 (28)	31.2±8.2 (8)	37.4±12.5 (2)	10.0±10.1	7.3–18.3
	Skokomish Hatchery	50.2±12.1 (11)	26.9±10.6 (0)	38.7±18.9 (0)	25.1±22.3 (0)	2.6±9.0	1.8–5.0

This model grouped wild Big Beef Creek and Skokomish River smolts as having different survival probabilities than Skokomish Hatchery smolts. Numbers of fish detected at the end of each segment are reported in parentheses.

a PR = Point of Release.

b RM = River Mouth.

c HCB = Hood Canal Bridge Line.

d ADM = Admiralty Inlet Line.

e JDF = Strait of Juan de Fuca Line.

f calculated using upper and lower 95% confidence limit detection probability estimates for the fixed JDF value in the model [31] (see methods section).



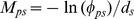



Where 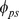 represents the survival probability for population *p* through segment *s*, and 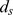
equals the distance between the first and last receiver line bounding segment *s*. Instantanous mortality rate expressions typically scale mortality rate by units of time (e.g., days, years), but here we use distance units (kilometers) due to the migratory behavior of steelhead smolts. Distance is a more appropriate scale for this application because smolts presumably covered similar migration distances while migration time was more variable. Population-specific and year-specific φ for calculations of 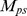
were derived from a model with a higher QAIC_c_ (in relation to the best model) that included the population and year variables (φ(segment × population + year), p(segment + RM:line).

### Migration Behavior Analysis

Each tagged fish was assigned a smolt index (SI), which characterized the extent to which an individual had undergone smoltification based on physical characteristics (1 = distinct parr marks, no silvering; 2 = some silvering, body elongation, parr marks still visible; or 3 = complete silvering, body elongation, parr marks no longer visible, and black fin margins, adapted from [31]). All wild smolts from the Skokomish River and Big Beef Creek, as well as hatchery smolts from the Duckabush River, were characterized with smolt indices of either two or three. However, several age-two hatchery smolts from the Skokomish River had smolt indices of one, indicating lack of readiness to migrate. We hypothesized that smolt index would predict migration success in these hatchery smolts. Counts of fish detected or not detected at the Skokomish River mouth with smolt indices of one were compared to counts of fish with smolt indices of two or three using a G-test of independence [32] to determine differences in estuary detection between groups.

Freshwater travel rate, marine travel rate, and migration range (distance between the Skokomish River estuary and the northernmost detection point) were calculated for each individual, but not all individuals were detected at locations necessary for parameter calculation. Range was calculated using the telemetry data analysis program AquaTracker (publicly available, contact: Jose.ReyesTomassini@noaa.gov). General linear models were used to test for statistical differences in wild and hatchery smolt parameters, with year included as a fixed factor. Interactions between year and rearing type were tested, and Tukey’s multiple comparison tests were carried out when interactions were significant (P≤0.05).

RM-HCB migration behavior of Skokomish populations was investigated using a plotting tool within AquaTracker that uses concentric circles of variable diameter to represent the proportion of tagged fish detected at each receiver deployed in 2008. Plots of hatchery and wild smolt detections from the Skokomish were compared to identify differences in distribution that may have affected survival within Hood Canal.

## Results

### Detection

The detection model with the lowest QAIC_c_ (*p*(segment + RM:line), Table 3) estimated separate RM detection probabilities for each river mouth and shared detection probabilities at the HCB, ADM, and JDF receiver lines over the three years, meaning there was not enough yearly variation to justify estimating separate parameters for each year. The model containing the same variables but with the addition of the release date covariate (*p*(segment + RM:line + rd)) had a similar QAIC_c_ (Δ0.118), indicating a possible effect of release date on detection probability. The Big Beef Creek RM line had the highest detection probability (92.8± SE 3.5%), while the Duckabush and Skokomish RM lines were less efficient (48.9±6.5% and 41.5±10.6%, respectively). The HCB (76.7±6.6%) and ADM (75.7±10.7%) probabilities were similar, and higher than the fixed JDF detection rate (68.5% based on [30]).

### Survival

**Figure 2:**
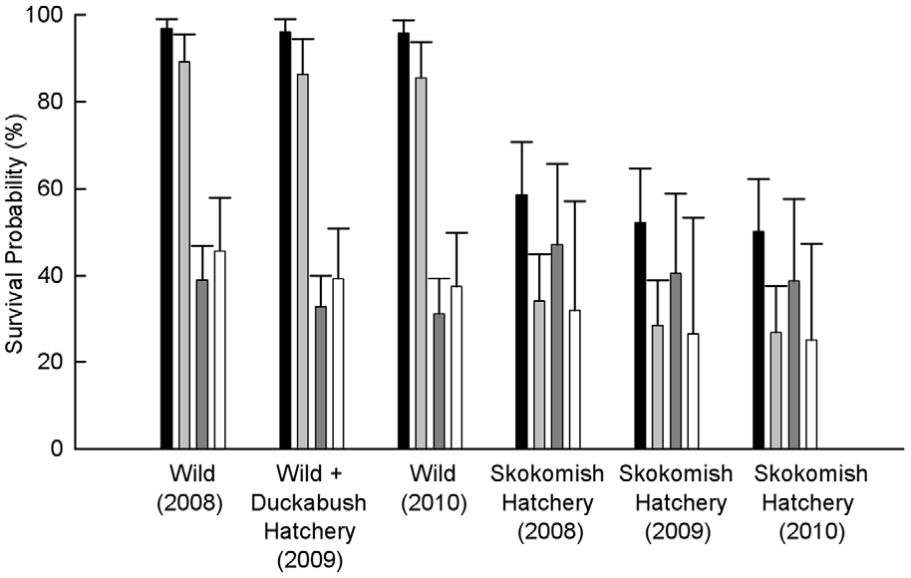
Survival estimates for smolts migrating through fresh- and saltwater migration segments. Survival probabilities (±SE) are derived from the survival model with the lowest QAICc that included year (φ(segment × SkokH + year + rd), *p*(RM:line + rd), which grouped survival probabilities for the wild Big Beef and wild Skokomish smolt groups and the Duckabush hatchery group and estimated the Skokomish hatchery group separately. Black bars represent freshwater survival probabilities, light gray bars represent river mouth to Hood Canal Bridge (RM-HCB) survival probabilities, dark gray bars represent Hood Canal Bridge to Admiralty Inlet (HCB-ADM) survival probabilities, and white bars represent Admiratly Inlet to Strait of Juan de Fuca (ADM-JDF) survival probabilities for smolts migrating in 2008, 2009, and 2010. Error bars reflect variation among years within each smolt group.

The survival model with the lowest QAIC_c_ included the interaction between segment and the SkokH variable and also included the rd covariate (φ(segment × SkokH + rd), Table 3). Length and condition factor were included in a similar model with a QAIC_c_ value only slightly higher than the model with the best fit (Δ2.27). Year was not included in the best model, though was included in a similarly ranked model (Δ2.67), indicating uncertainty about the effect of yearly variation on estimated survival of Hood Canal steelhead. The interaction between segment and SkokH indicates that the Skokomish hatchery fish survival probabilities differed from survival probabilities of other populations for some but not all segments. Specifically, Skokomish hatchery smolts had lower survival probabilities than all other populations within all except the HCB-ADM migration segment (Figure 2, Table 4). In contrast, the Duckabush hatchery smolts experienced survival probabilities similar to the two wild populations, as indicated by the “SkokH” grouping factor in the model with the lowest QAIC_c_. (Table 4).

**Figure 3:**
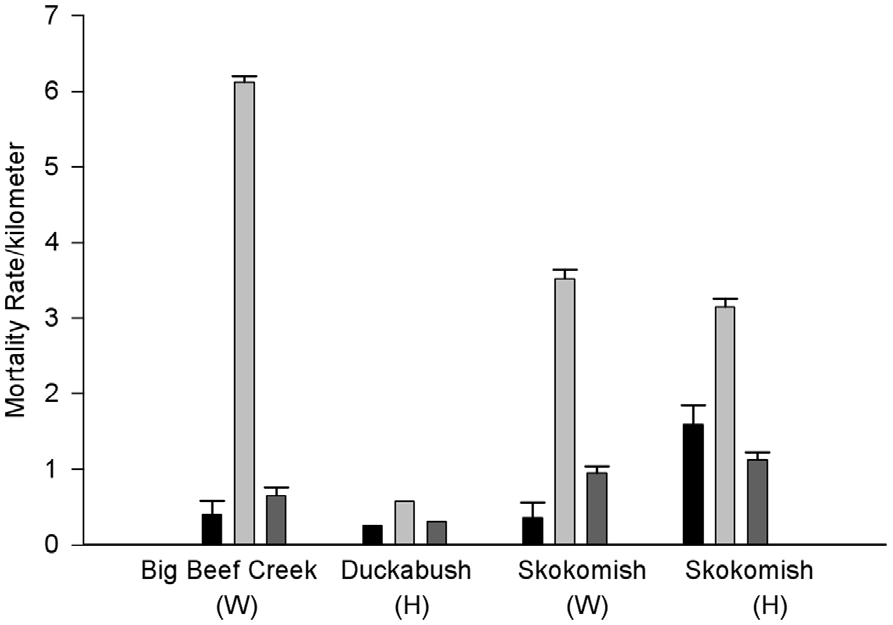
Distance-based instantaneous mortality rate for marine migration segments. Segment- and year specific instantaneous mortality estimates for each tagged group were scaled by the distance of each segment in kilometers (km). Mean rates for 2008, 2009, and 2010 (±SE) are presented by population. Duckabush population rate is for 2009 only. Black bars represent RM-HCB mortality rate, light gray bars represent HCB-ADM mortality rate, and dark gray bars represent ADM-JDF mortality rate.

Several spatial and temporal patterns can be identified among migration segments and years. Wild and Duckabush hatchery populations experienced the highest survival probabilities in freshwater and inside the Hood Canal, then experienced lower HCB-ADM and ADM-JDF survival probabilities (Table 4). The Skokomish hatchery population tended to experience low PR-RM, RM-HCB, and ADM-JDF survival probabilities and relatively high survival probabilities from HCB-ADM (Figure 2, Table 4). All populations experienced the highest instantaneous mortality rates per unit of distance between the HCB and ADM lines (Figure 3). The effect of year was not an obvious source of variation in the data, but the linear pattern imposed by the structure of the model with the lowest QAIC_c_ that did include year suggests a negative trend over time, with RM-JDF survival estimated to be highest in 2008 (Table 4). Release date was included as a coviariate in the survival model with the lowest QAIC_c_. The importance of considering release date in survival estimation is increased by it’s inclusion in all 32 models with the lowest QAIC_c_ values (Table 3). The release date beta estimate is positive (0.027), indicating a slight increase in survival rate for fish with later release dates. However, release date may be somewhat confounded with population, since Duckabush Hatchery smolts were released slightly later than the other populations (Table 1).

### Migration Behavior of Skokomish smolts

The likelihood of detecting Skokomish hatchery fish at the river mouth depended on smolt index (G_adj_ = 7.149, df = 1, p = 0.004). Only 7% of fish with smolt indices of one were detected at or beyond the river mouth, whereas 69% of fish with indices of two or three were detected at the river mouth or on a marine receiver. Though mortality and lack of migration are indistinguishable using telemetry methods, these results suggest that some Skokomish hatchery fish are residualizing, or failing to migrate to saltwater.

**Figure 4:**
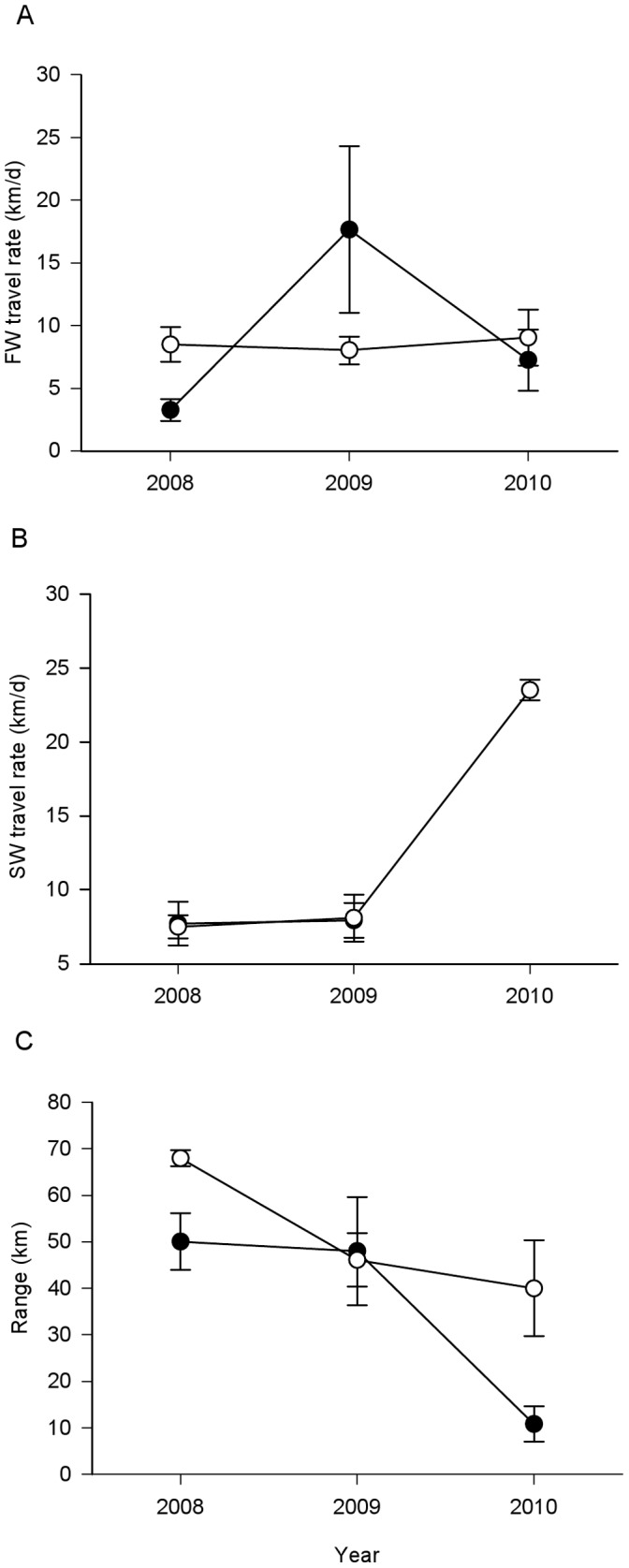
Migration behavior of wild and hatchery Skokomish smolts. (A) Mean travel rate (±SE) from the point of release to the river mouth (13.5 km) for hatchery (black circles) and wild (open circles) individuals from the Skokomish River. (B) Mean travel rate (±SE) from the river mouth to the Hood Canal Bridge (75 km) for hatchery (black circles) and wild (open circles) individuals from the Skokomish River. (C) Mean migration range (distance between the Skokomish River estuary and the northernmost detection point) (±SE) for hatchery (black circles) and wild (open circles) individuals from the Skokomish River. Black circles are partially obscured by the white circles.

Effects of rearing type on freshwater travel rate differed among years (GLM; rearing type: F_1,57_ = 3.75, p = 0.058, year: F_2,57_ = 4.45, p = 0.016, rearing type × year; F_2,57_ = 3.62, p = 0.033; Figure 4a). Mean hatchery fish freshwater travel rates were slower than mean wild fish freshwater travel rates in 2008 and 2010, but faster in 2009; however, the post-hoc Tukey’s tests did not indicate significant differences within any year (all P>0.280).

Saltwater travel rates were not significantly different between hatchery and wild populations, and rates for both smolt groups did not vary significantly among years (GLM; rearing type: F_1,57_ = 0.00, p = 0.993, year: F_2,57_ = 1.55, p = 0.223; Figure 4b).

Wild Skokomish smolts displayed significantly greater ranges of detection in Hood Canal than did their hatchery counterparts (GLM; rearing type: F_1,96_ = 10.20, p = 0.002, year: F_2,96_ = 17.94, p<0.001, rearing type × year; F_2,96_ = 3.17, p = 0.047; Figure 4c). On average, wild smolts progressed farther in their migration toward the open ocean than did hatchery fish in 2008 (Tukey’s Multiple Comparison; P = 0.043) and 2010 (P = 0.032), but not in 2009 (P = 0.999).

**Figure 5:**
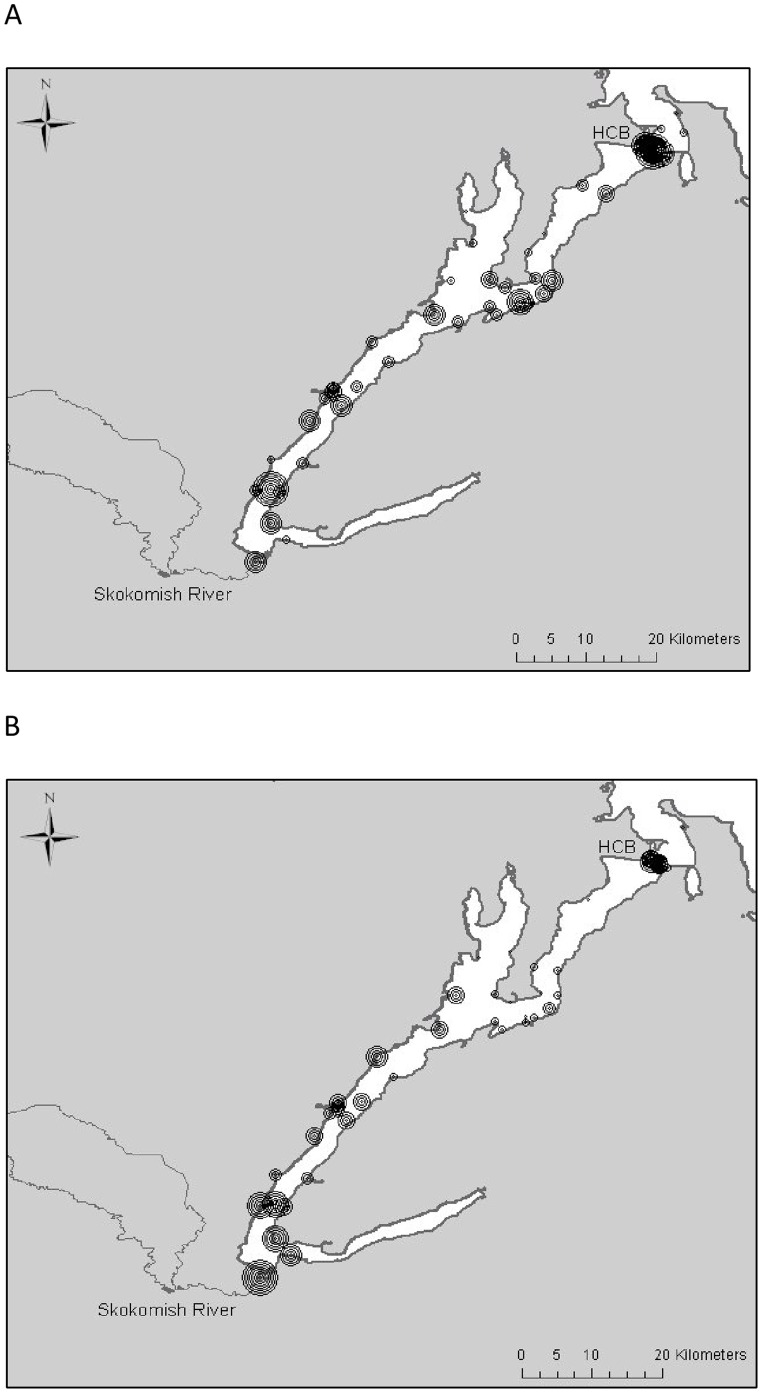
Density plots of wild and hatchery Skokomish smolt migration in 2008. Concentric circles represent the relative number of smolts detected at each acoustic receiver. Larger circles represent a greater proportion of the total number of fish detected in the Hood Canal (8 concentric circles = 50%) than smaller circles, which represent a small proportion (1 circle = 10%) of the fish in the sample. Plot (A) depicts 2008 Skokomish wild smolt detection densities, and plot (B) shows 2008 Skokomish hatchery smolt detection densities.

The distribution plots corroborated the results observed in the analysis of migration range, showing many more wild smolts detected toward the northern outlet of Hood Canal compared to hatchery smolt detections (Figure 5).

## Discussion

Soon after Pacific salmon and steelhead enter the marine environment, mortality rates exceed those incurred during later periods of marine residency [21], [33], [34]. Whether related to predation [35], a growth imperative [23], or both [36], low rates of early marine survival likely play a prominent role in limiting adult salmon and steelhead returns. Wild steelhead smolt survival probabilities through Hood Canal only (RM-HCB) measured in 2006 and 2007 (62.0–85.6%; [21]) are in agreement with 2008–2010 survival probabilities through the same segment (85.5–89.2%). In contrast, wild smolt survival probabilities from marine entry through the last detection array (RM-JDF) ranged from 10.0–15.9% for 2008–2010 outmigrants, which were substantially lower than the estimates for wild Hood Canal smolts in 2006 (28.6–41.7%; [21]) and lower than estimates of wild smolts migrating longer distances through the Georgia Basin in 2004–2006 (18–45%; [29]). Moore et al. [21] used more powerful (Vemco V9) transmitters in 2006 and assumed a 100% detection probability at the JDF line to estimate RM-JDF survival in that year. The present study used a fixed detection probability inferred from the probability calculated by Melnychuck [30] at a similar receiver line (Strait of Georgia line) in 2005 through 2007. We used the upper and lower 95% confidence level values to compute a range of survival estimates for each population to estimate variation in 2008–2010 RM-JDF survival. The 2010 HCB-ADM and ADM-JDF survival probabilities were low relative to both the 2006 HCB-JDF estimates [21] and the HCB-ADM and ADM-JDF from the previous two years, which may reflect a negative trend in survival over time (see Table 4).

Other species of Pacific salmon (ocean-type Chinook, chum, and pink) smolts are prohibitively small for acoustic tagging studies, but Welch et al. [29] showed that Georgia Basin wild sockeye salmon smolts survive the early marine residence period with probabilities similar to those of steelhead (25–30% for 2005 outmigrants). An acoustic telemetry study in a tributary of the Fraser River in British Columbia found that coho salmon (mixture of hatchery, wild, and hatchery/wild hybrid; N = 8) smolts survived the early marine period at a minimum rate of 25%, although a significant proportion of coho were suspected to have remained in freshwater [37]. The survival probabilities reported in the present study and cited above are the first estimates of early marine survival in the Salish Sea, and there are no historical rates to serve as comparison. Smolt-to-adult survival rates of steelhead originating in southern British Columbia and Washington inland rivers (i.e., rivers feeding into the Strait of Georgia, Johnstone Strait, and Puget Sound) declined significantly in the early 1990’s and have remained low [38], [39]. Although no comparable early marine survival estimates are available, the growing evidence of low steelhead survival in the Salish Sea [21], [40], [41] appears to be contributing to the lower smolt-to-adult survival rates observed over the last couple of decades.

Distance-based instantaneous mortality rate between two consecutive receiver lines varied among the migration segments. An insignificant portion of mortality occurred during the short (0.05–13.5 km) freshwater migration segment for the wild and Duckabush hatchery populations. Mortality rates within Hood Canal and between the ADM and JDF lines were similar. All populations experienced mortality rates between the HCB and ADM lines that were two to fifteen times greater than the other two marine migration segments. We hypothesize disruption in migration caused by the Hood Canal Bridge and associated increases in predation risk explain at least some of the elevated mortality in the HCB to ADM segment (discussed in [21]). Shading caused by overhead structures has been associated with a change in behavior of juvenile salmon, which tended to school more frequently and avoided occupying such shaded habitat [42]. Marine mammal predators have been shown to specifically target similar congregations of adult salmon when anthropogenic barriers alter natural migration patterns (e.g., Ballard Locks [43] or Bonneville Dam [44]). Shade cast by the Hood Canal Bridge may affect juvenile salmon and steelhead migration. Submerged concrete floating pontoons on the Hood Canal Bridge extend 3.6 meters underwater and may exacerbate behavioral abnormalities as surface-oriented steelhead must navigate around or under the in-water structures.

The efficacy of using conservation hatcheries to rebuild diminished salmon and steelhead populations depends largely on achieving high post-release survival. Our results demonstrate that hatchery-reared smolts can achieve early marine survival probabilities similar to wild smolts in the same migratory corridor, and that migration performance may strongly depend on the hatchery conditions and practices employed during one or two years of captivity. Skokomish hatchery smolts survived poorly in the freshwater and marine environment compared to wild Skokomish and wild Big Beef Creek smolts, while Duckabush hatchery smolts survived as well or better than wild populations. Moore et al. [21] found that Hamma Hamma River (12.5 km south of the Duckabush River) hatchery smolts raised at the Lilliwaup Hatchery and released in 2006 and 2007 performed within the range of co-migrating wild smolts through most migration segments. These Hamma Hamma hatchery smolts survived at rates similar to those estimated for Duckabush hatchery smolts in 2009, supporting the results of this study. Johnson et al. [45] measured survival rates of one wild and two hatchery steelhead smolt groups migrating through the Alsea River and estuary in Oregon, and found no significant effects of either hatchery treatment (hatchery smolts were offspring of either wild or domesticated broodstock). Kostow [2] observed contrasting results, observing much higher smolt-to-adult survival rates for wild steelhead (5-year mean = 5.62%) compared to “new” (offspring of mostly wild broodstock; 5-year mean = 0.9%) and “old” (offspring of domesticated, non-local broodstock; 5-year mean = 1.1%) hatchery steelhead groups. Results of survival studies comparing hatchery and wild steelhead vary widely, which may be resultant of the considerable variation in rearing strategies across studies. The survival differences between hatchery groups and between wild and hatchery Skokomish smolt survival observed in this study suggest some aspects of rearing conditions or practices at the McKernan hatchery were not promoting optimal post-release migration and survival.

Despite some important similarities between hatcheries (water temperature, food ration, food type), rearing conditions differed in at least three specific ways: (1) fish at Lilliwaup were held at lower densities than those at McKernan, (2) fish were size sorted twice at Lilliwaup and only once at McKernan to decrease variability in fish size within a vessel, and (3) Lilliwaup fish were kept in circular tanks thoughout time in captivity while fish at McKernan were transferred to a 6.7 m × 44.8 m raceway at age-1 (Table 2).

Negative effects of high hatchery rearing densities on behavior and physiology of salmonids include impaired competitive ability in captively-reared brown trout [46] and reduced ability to locate food, recognize and ingest novel food types, avoid predators, and survive under natural conditions [47]. Studies conducted at several Chinook salmon hatcheries in the Columbia River Basin agree that marine survival is highest for fish raised at densities substantially lower than maximum hatchery carrying capacity [48]. Mean densities are variable among traditional Puget Sound *O. mykiss* hatcheries; density indices generally range from 0.025–0.95 lbs/ft^3^/inch fish length (Puget Sound HGMP documents available from http://www.nwr.noaa.gov/Salmon-Harvest-Hatcheries/Hatcheries). Density indices were extremely low at Lilliwaup Hatchery (year one: 0.002–0.008 lbs/ft^3^/inch, year two: 0.004–0.010 lbs/ft^3^/inch), and were higher and within the Puget Sound traditional hatchery range at McKernan Hatchery (year one: 0.047–0.778 lbs/ft^3^/inch, year two: 0.047–0.065 lbs/ft^3^/inch). Densities similar to those maintained at the Lilliwaup Hatchery may not be feasible for augmentation hatcheries, but the improvement in natural behavior and survival associated with low density rearing suggests a potentially worthwhile trade-off for conservation programs.

Tank volume and geometry differed between hatcheries, and may have compounded the effects of increased rearing density to affect behavior and survival of hatchery steelhead in this study. During the second year of rearing, McKernan Hatchery fish were reared in one large raceway, while the Lilliwaup Hatchery used three 6-m diameter circular tanks, which offer more structure and shade (from tank walls) per unit of volume than the raceway provided. Casual observations made by hatchery staff suggest that fish reared in the raceway tend to crowd together in turbulent water areas and against the walls of the raceway, further increasing the effective density (fish per unit volume), whereas circular tanks promoted a more uniform distribution of fish within each rearing vessel. We hypothesize that the marked differences in rearing density, and perhaps compounding effects of tank geometry on crowding behavior, influenced important parameters of fish health and condition, and ultimately their post-release survival.

Hatchery environments obviously differ from natural environments experienced by wild fish, and whether through domestication selection or developmental factors, fish raised in captivity are generally phenotypically and behaviorally different than wild counterparts [49]. However, hatchery environments can vary substantially and rearing conditions can be manipulated to substantially alter important parameters (e.g., density). The two hatchery populations in this study survived at significantly different probabilities through freshwater and the early marine migration period despite both hatchery groups experiencing very similar water temperatures, rations, and feed composition. In fact, variation in behavior and survival between hatcheries exceeded variation among the wild populations in this study and in the previous study [21]. Our results highlight the need for conservation hatchery programs, in particular, to identify and implement rearing practices that promote natural behavior and high post-release survival to benefit target populations.
